# MANAGING MULTIPLE CHRONIC CONDITIONS: FINDINGS BY NATIONAL CLAUDE D. PEPPER OLDER AMERICANS INDEPENDENCE CENTERS

**DOI:** 10.1093/geroni/igae098.2175

**Published:** 2024-12-31

**Authors:** Eileen Graham, Rachel O’Conor, Michael Wolf

**Affiliations:** Chair; Co-Chair; Discussant

## Abstract

Multimorbidity is an increasing concern among clinicians, as older adults accumulate chronic conditions that require complex coordination of care. There is an increasing interest among research and clinical communities to understand the impacts of multimorbidity on individuals and how quality of care can be optimized to improve quality of life in the aging population. The primary objective of the Claude D. Pepper Older Americans Independence Centers national network is to advance scientific knowledge that will help older adults maintain function, independence, and quality of life. The current symposium highlights recent work put forth by three of the 15 National ‘Pepper’ Centers (Northwestern, Mt. Sinai, UCSF). Graham will present recent findings on how multimorbidity is longitudinally related to physical and mental health-related quality of life, and their cognitive, psychological, social, and health-related determinants. The next three presentations explore the role of caregivers in supporting the health and quality of life of older adults with multimorbidity. O’Conor will discuss complexity of family care dyads management of MCC, by reviewing patient and caregiver perspectives on challenges of successful management at home. Allison will discuss the interplay of care support structures and dementia care needs in the home. Reckrey will discuss results from a qualitative analysis exploring perspectives of geriatricians on the importance of paid caregivers of older adults with MCC. In sum, this symposium will present novel evidence showing the importance of understanding of how multimorbidity and dementia impact caregiving considerations, primary care decisions, and health related quality of life for older adults.

